# Supplementary material for: Metagenomic Assembled Genomes of a Pseudanabaena Cyanobacterium and Six Heterotrophic Strains from a Xenic Culture
Source: Microorganisms. 2025 Aug 27;13(9):1996. doi: 10.3390/microorganisms13091996 (PMC12472891; doi:10.3390/microorganisms13091996)
Supplement: Supplementary file 1 [file microorganisms-13-01996-s001.zip › SI_File_1.pdf]

Article

Metagenomic Assembled Genomes of a *Pseudanabaena* Cyanobacterium and Six Heterotrophic Strains from a Xenic Culture

Paul D. Boudreau

Department of BioMolecular Sciences, University of Mississippi School of Pharmacy, Oxford, MS 38677, USA; boudreau@olemiss.edu

Keywords: cyanobacteria; metagenomics; microbiome; whole genome sequencing

Contents

1. Materials and Methods Supplement . . . 2
   1.1. General Experimental Procedures and Details . . . 2
      1.1.1. Equipment Used . . . 2
2. PCR Primers and Methods . . . 2
3. Phylogenetic Trees . . . 3
4. Read and Genome Statistics . . . 10
5. References . . . 11

Received:
Revised:
Accepted:
Published:

Citation: Lastname, F.; Lastname, F.; Lastname, F. Title. *Microorganisms* **2025**, *1*, 0. https://doi.org/

Copyright: © 2025 by the authors. Submitted to *Microorganisms* for possible open access publication under the terms and conditions of the Creative Commons Attri- bution (CC BY) license (https://creativecommons.org/licenses/by/4.0/).

1. Materials and Methods Supplement

1.1. General Experimental Procedures and Details

1.1.1. Equipment Used

The thermal cycler program for these PCR reactions was run with an Analyik Jena Biometra TOne 96G Thermal Cycler. The gel electrophoresis system was a VWR Electro-phoresis System Mini 115V. DNA was quantified with a Qubit 4 Fluorometer using the Qubit 1x dsDNA HS Assay Kit (Invitrogen). The LED grow lights were from Gardner's Supply Company. The freezers used were a VWR Ultra-Low Temperature Upright Freezer set to -70 °C or a Thermal-Kool walk in freezer set below -20 °C. the heat block used was a Fisherbrand Mini Dry Bath. Centrifuges were a Sorvall Legend Micro 21R Microcentrifuge and a Sorvall Legend XTR Fixed Angle Centrifuge. The freeze dryer was a Labconco Freeze Dry System/Freezone 2.5. The vortexer was a Fisherbrand Vortex Genie 2. The gel electro-phoresis was run with a VWR Electrophoresis Mini 115V System.

2. PCR Primers and Methods

Table S1. Primers Used in Amplicon Sequencing.

Primer Name	Sequence (5′ and 3′)	Reference
TS19	GGGGAATTTTCCGCAATGGG	Boyer et al., 2021 [1]
TS26	TCAGCCTGTTATCCCTAGAG	Sherwood et al., 2007 [2]
PB10	CGTCGTGGCGAATTCAAGTC	This work
PB13	GTCCACCAATACCTGCACCA	This work
PB15	TCAGATCCGCTTGATACGCC	This work
PB16	GTGAGGATTGGTTCAGCGCT	This work

Table S2. Thermal Cycler PCR Methods.

Method for the rRNA Amplicon		
**Step**	**Temperature (°C)**	**Time (s)**
1	98	30
2	98	5
3	57	10
4	72	120
Return to Step 2 (x35 cycles)		
5	72	120
6	4	Hold
Method for Targeted Amplicons		
1	95	30
2	95	5
3	57	10
4	72	480
Return to Step 2 (x35 cycles)		
5	72	120
6	8	Hold

3. Phylogenetic Trees

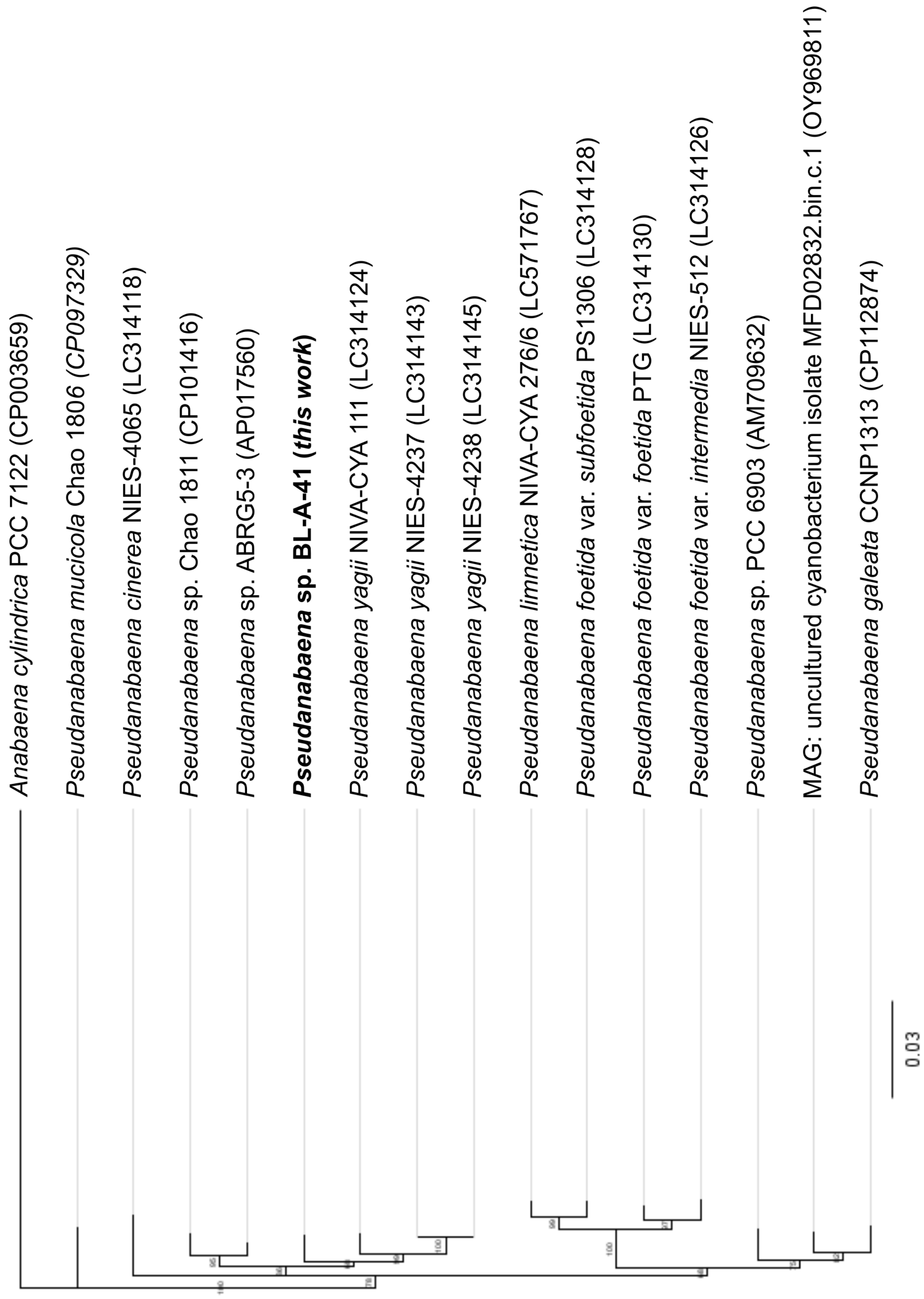

Figure S1. Ribosomal Region Phylogenetic Tree for *Pseudanabaena* sp. BL-A-41.

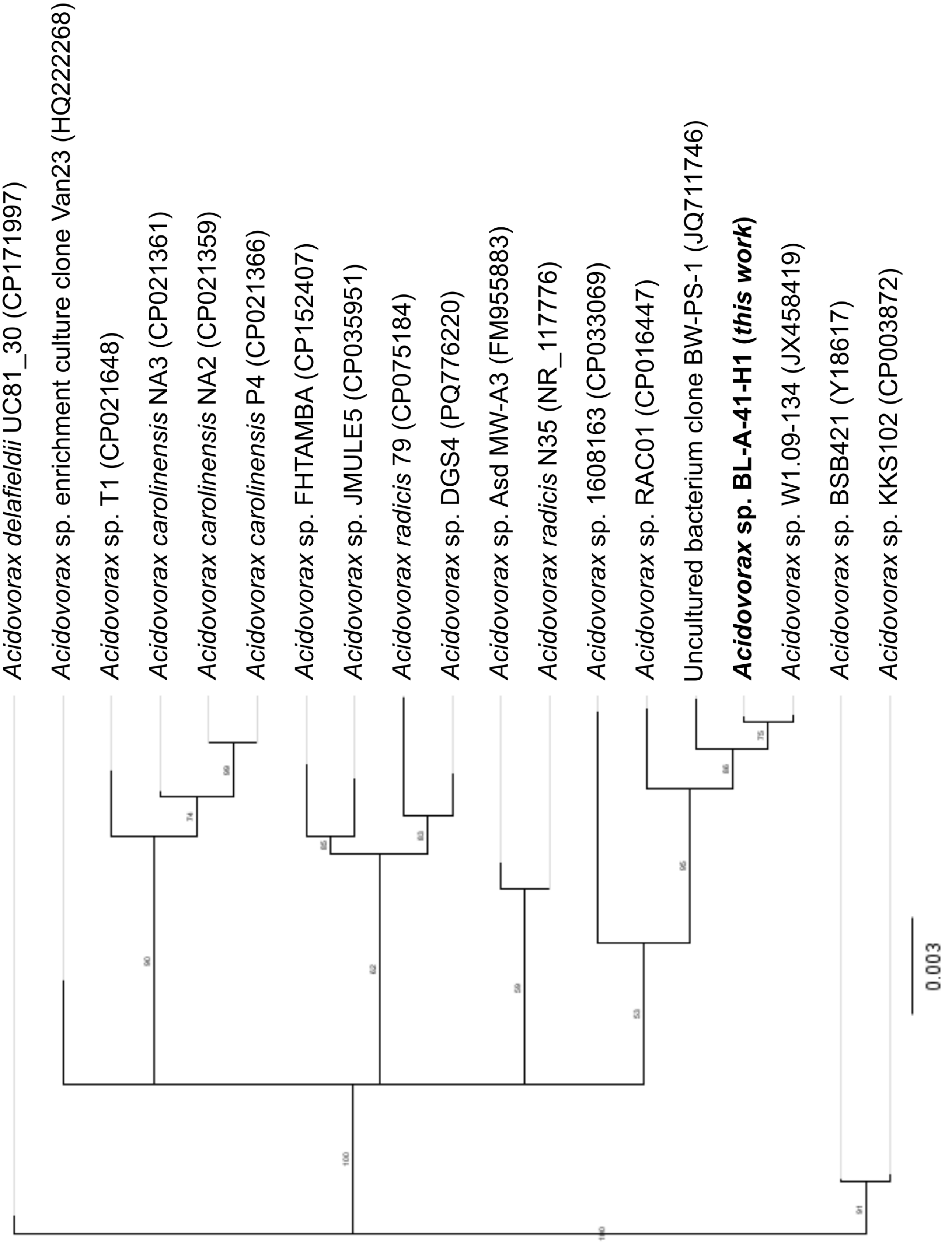

Figure S2. Ribosomal Region Phylogenetic Tree for *Acidovorax* sp. BL-A-41-H1.

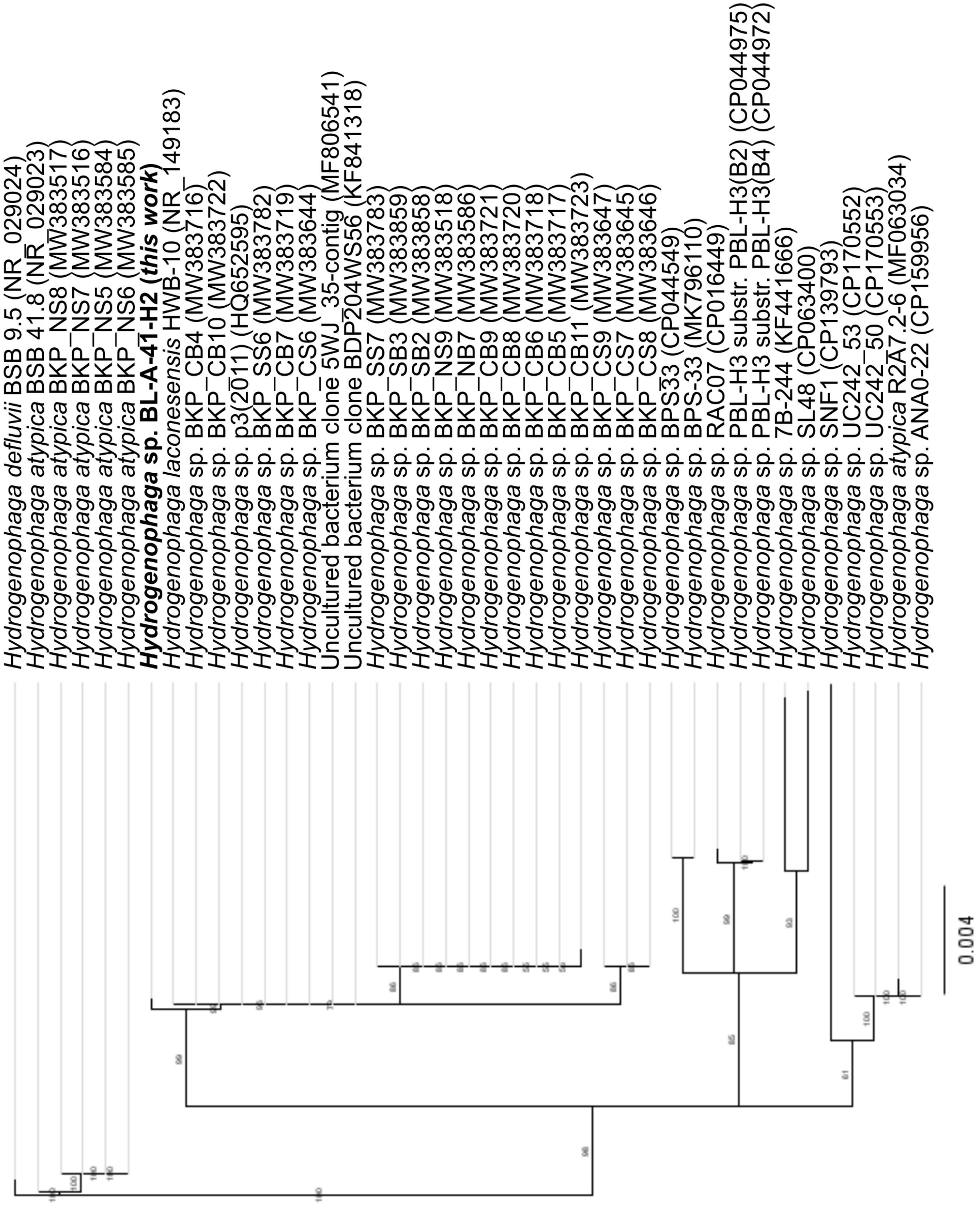

Figure S3. Ribosomal Region Phylogenetic Tree for *Hydrogenophaga* sp. BL-A-41-H2.

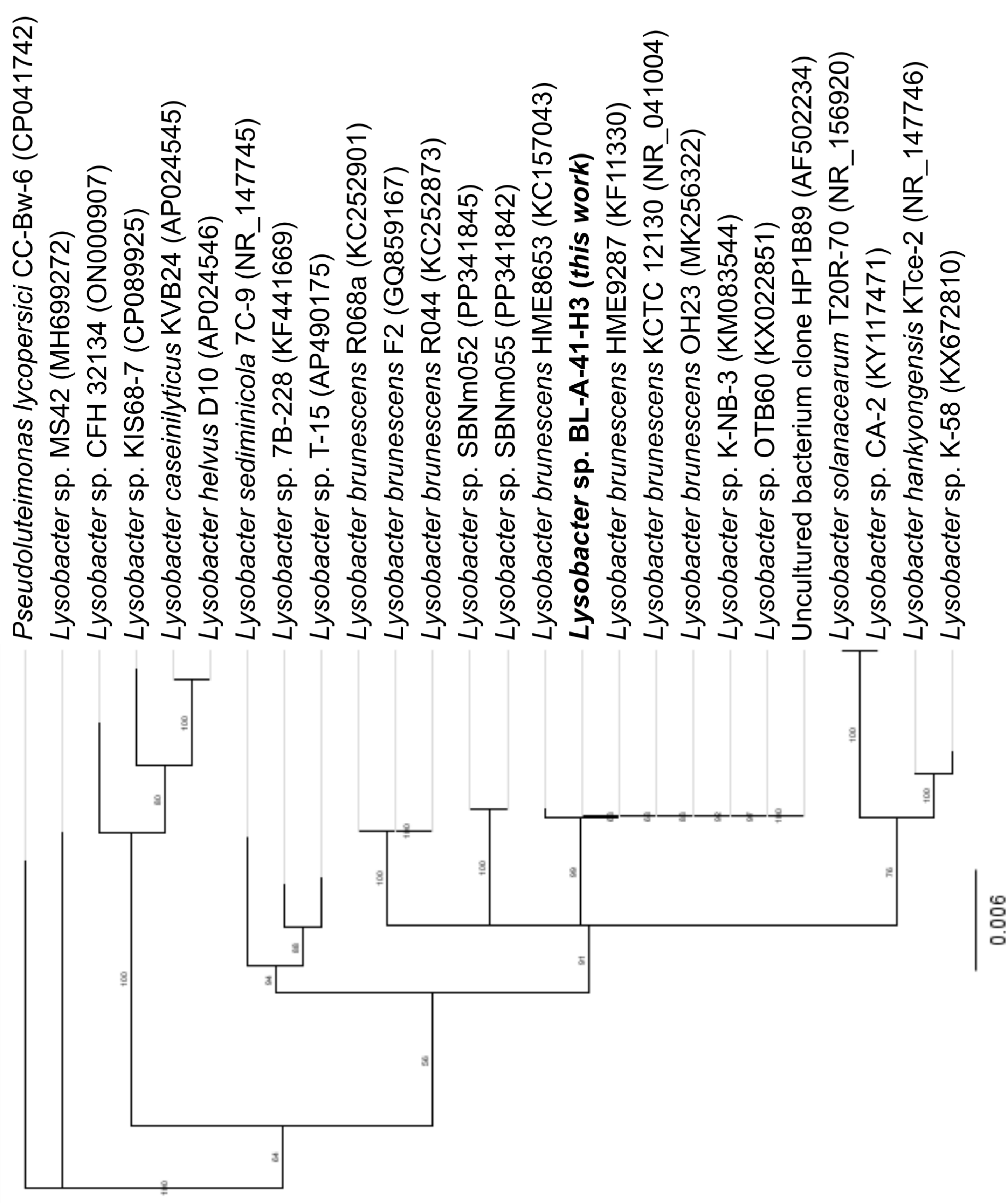

Figure S4. Ribosomal Region Phylogenetic Tree for *Lysobacter* sp. BL-A-41-H3.

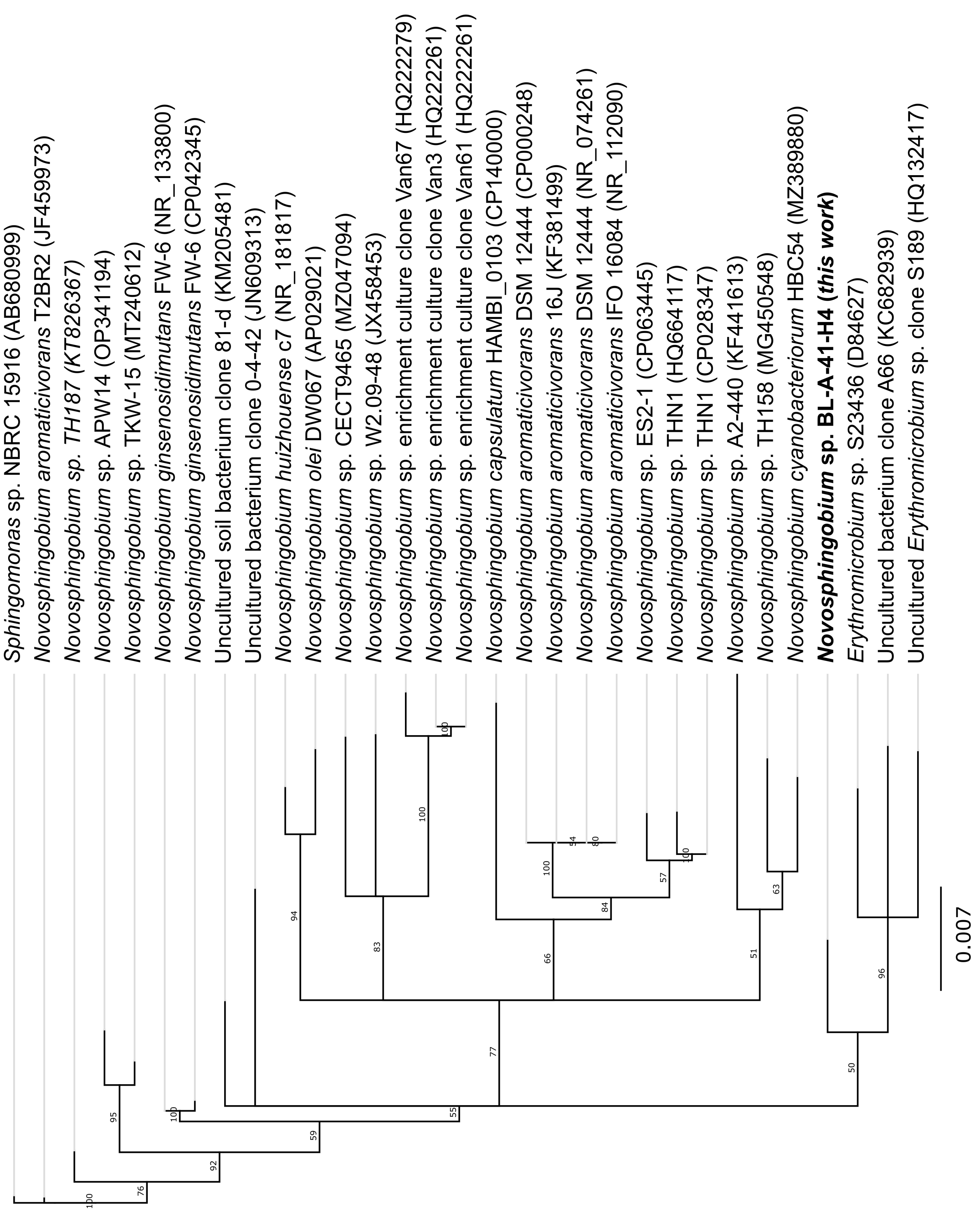

Figure S5. Ribosomal Region Phylogenetic Tree for *Novosphingobium* sp. BL-A-41-H4.

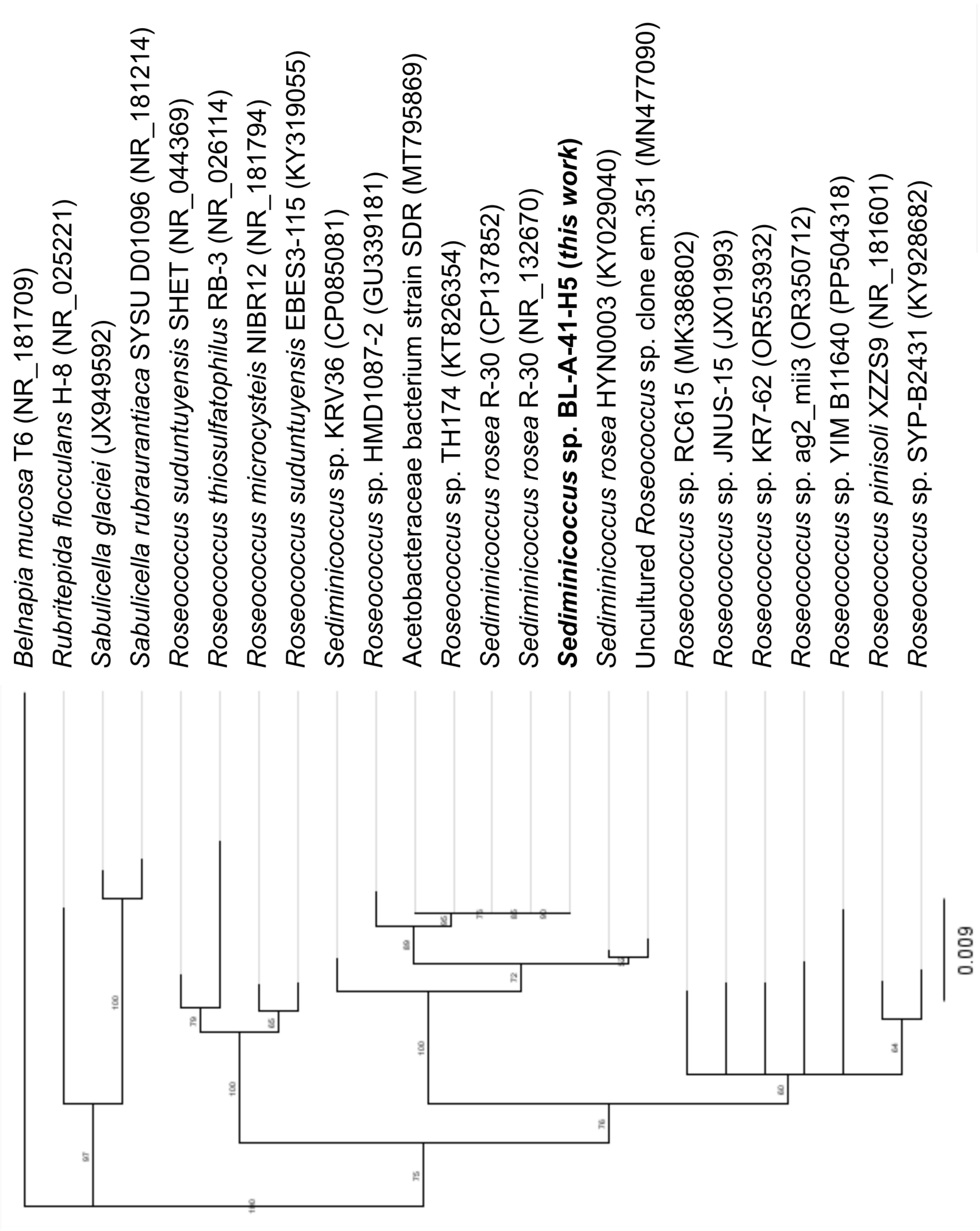

Figure S6. Ribosomal Region Phylogenetic Tree for *Sediminicoccus* sp. BL-A-41-H5.

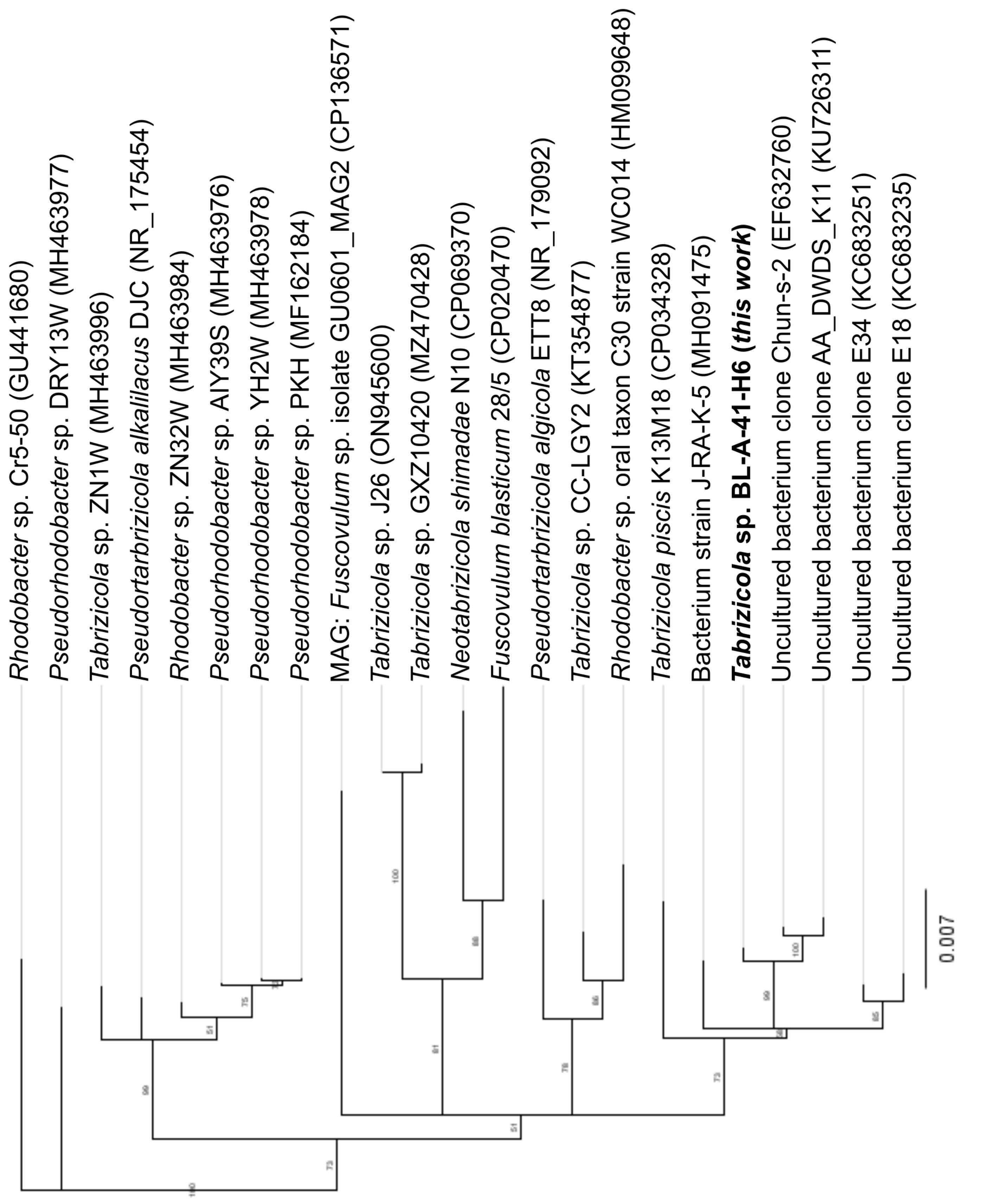

Figure S7. Ribosomal Region Phylogenetic Tree for *Tabrizicola* sp. BL-A-41-H6.

4. Read and Genome Statistics

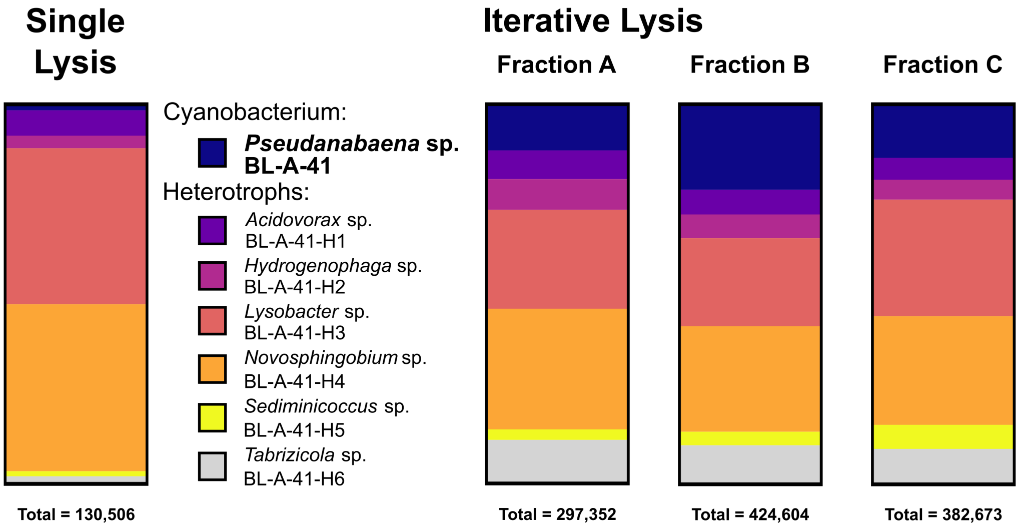

Figure S8. Read Origin as Determined by Geneious Mapping.

Table S3. Read Statistics of Sequencing Runs.

Read Set	Initial Reads		Post FiltLong at 500 bp and 99%		Post FiltLong at 1,000 bp and 90%	
	No. of Reads	N50 (bp)	No. of Reads	N50 (bp)	No. of Reads	N50 (bp)
Single Extraction	237,690	2,746	184,503	2,855	130,032	3,171
Fraction A	612,926	2,152	465,410	2,263	297,976	2,621
Fraction B	830,966	2,367	650,437	2,477	426,824	2,860
Fraction C	793,487	2,493	593,189	2,635	384,944	3,057
Pooled Frac. B and C	400,809	2,390	247,613	2,610	160,407	3,026

Table S4. Genome Statistics of Assembled Genomes.

Strain	Genome Size (Mb)	No. of Contigs	Fold Coverage	GC Content (%)	Completeness / Contamination* (%)
Pseudanabaena sp. BL-A-41	5.65	4**	111	42.5	98.96 / 1.09
Acidovorax sp. BL-A-41-H1	5.92	15	36	65.3	96.55 / 29.31
Hydrogenophaga sp. BL-A-41-H2	3.28	1	57	68.4	98.28 / 0.0
Lysobacter sp. BL-A-41-H3	4.64	1***	264	67.9	100.0 / 0.0
Novosphingobium sp. BL-A-41-H4	3.02	3	431	66.1	100.0 /0.0
Sediminicoccus sp. BL-A-41-H5	4.80	1	35	70.0	100.0 /0.0
Tabrizicola sp. BL-A-41-H6	4.10	1	97	64.1	98.28 / 0.0

* From DFAST via CheckM [3]

** One circular chromosome and three circular plasmids.

*** One circular chromosome.

References

1. Boyer, S.L.; Flechtner, V.R.; Johansen, J.R. Is the 16S–23S rRNA internal transcribed spacer region a good tool for use in molecular systematics and population genetics? A case study in cyanobacteria. *Mol. Bio. Evol.* **2001**, *18*, 1057–1069.
2. Sherwood, A.R.; Presting, G.G. Universal primers amplify a 23S rDNA plastid marker in eukaryotic algae and cyanobacteria 1. *J. Phycol.* **2007**, *43*, 605–608.
3. Tanizawa, Y.; Fujisawa, T.; Nakamura, Y. DFAST: a flexible prokaryotic genome annotation pipeline for faster genome publication. *Bioinformatics* **2018**, *34*, 1037–1039.

Disclaimer/Publisher's Note: The statements, opinions and data contained in all publications are solely those of the individual author(s) and contributor(s) and not of MDPI and/or the editor(s). MDPI and/or the editor(s) disclaim responsibility for any injury to people or property resulting from any ideas, methods, instructions or products referred to in the content.
